# Clinicopathological Significance of MicroRNA-20b Expression in Hepatocellular Carcinoma and Regulation of HIF-1*α* and VEGF Effect on Cell Biological Behaviour

**DOI:** 10.1155/2015/325176

**Published:** 2015-11-03

**Authors:** Tong-min Xue, Li-de Tao, Miao Zhang, Jie Zhang, Xia Liu, Guo-feng Chen, Yi-jia Zhu, Pei-Jian Zhang

**Affiliations:** ^1^Institute of General Surgical Research, Second Affiliated Hospital, Yangzhou University, Yangzhou, Jiangsu 225002, China; ^2^Nanjing University of Chinese Medicine, Nanjing, Jiangsu 210023, China

## Abstract

miRNA-20b has been shown to be aberrantly expressed in several tumor types. However, the clinical significance of miRNA-20b in the prognosis of patients with hepatocellular carcinoma (HCC) is poorly understood, and the exact role of miRNA-20b in HCC remains unclear. The aim of the present study was to investigate the association of the expression of miR-20b with clinicopathological characteristics and overall survival of HCC patients analyzed by Kaplan-Meier analysis and Cox proportional hazards regression models. Meanwhile, the HIF-1*α* and VEGF targets of miR-20b have been confirmed. We found not only miR-20b regulation of HIF-1*α* and VEGF in normal but also regulation of miR-20b in hypoxia. This mechanism would help the tumor cells adapt to the different environments thus promoting the tumor invasion and development. The whole study suggests that miR-20b, HIF-1*α*, and VEGF serve as a potential therapeutic agent for hepatocellular carcinoma.

## 1. Introduction

Hepatocellular carcinoma (HCC) represents an extremely poor prognostic cancer that remains one of the third causes of cancer-related death and aggressive human malignancies represented worldwide [1, 2]. The dismal outcome has been attributed to the major hallmarks of HCC, intrahepatic metastases or postsurgical recurrence [3]. With much progress made in surgery and other treatments, the prognosis of HCC patients is still unsatisfactory due to the high rate of recurrence and metastasis. Thus, it is crucial to improve our understanding of the molecular mechanisms underlying HCC which will be critical for the improvement of therapeutic strategies for HCC patients [4]. However, tumor metastasis is considered to be one of the most complex cell activities because it is a multistep process of cascade involving cell invasion and intramedullary pin into the blood and lymph vessels, survival and arrests in the vascular system, the proliferation of extravasation.

MicroRNAs (miRNAs) are a class of small noncoding RNA molecules that regulate gene expression by binding to partially complementary recognition sequences of target mRNAs, either repressing miRNA translation or cleaving target miRNA and RNAs that are highly conserved between species [5–8]. In recent years, increasing studies indicate that miRNAs have crucial functions in specific cellular processes such as differentiation, morphogenesis, and tumorigenesis and they are also considered as oncogenes and tumor suppressors [9–12]. Recently, it has been manifested that the deregulation or dysfunction of miRNAs is involved in cancer development and related to clinical outcomes of cancer patients including HCC [13]. In HCC, miRNAs have been discovered to be aberrantly expressed and some of them are functionally involved in HCC carcinogenesis, progression, and metastasis [14, 15]. However, the roles of a large number of miRNAs are still unexplored in HCC [4].

Hypoxia inducible factor-1 (HIF-1) is the first identified mediator of cell response to hypoxia in mammalian cells cultured under reduced oxygen tension [16]. The transcription factor HIF-1 consists of HIF-1*α* and HIF-1*β* and is a key regulator responsible for the induction of genes that facilitate adaptation and survival of tumor cells from hypoxic microenvironment and confer on the tumor a worse malignant phenotype [17, 18]. The overexpression of HIF-1*α* was found in various types of cancers of both human and mouse [17, 19]. The HIF-1*α* complex acts as a transcription factor for many target genes in several aspects of cancer progression including angiogenesis, glucose metabolism, cell proliferation, and apoptosis [20, 21]. Vascular endothelial growth factor (VEGF) is one of the major target genes for HIF-1*α* that directly participates in angiogenesis and a recognized therapeutic target [22, 23]. VEGF, the most potent angiogenic molecule, participates specifically in promoting vascular endothelial cell division, proliferation, and migration [24].

Some studies have shown that miRNA-20b is deregulated in several types of cancers [25–29] and upregulation of miRNA-20b correlates with worse prognosis; all these studies indicated that miRNA-20b acts as a tumor promoter [28, 30–34]. Some studies have shown that miRNA-20b modulates HIF-1*α* and VEGF to keep tumor adapting to different environment and promoting cell division, proliferation, and migration [27, 33, 34]. At present, the clinical significance of miRNA-20b in the prognosis of patients with HCC is poorly understood, and the exact role of miRNA-20b in HCC remains unclear. Here, we investigated the association between miRNA-20b expression and clinicopathological parameters and assessed the effect of miRNA-20b modulating HIF-1*α* and VEGF on biological behaviours including cell proliferation, apoptosis, and migration of HepG2 cells.

## 2. Materials and Methods

### 2.1. Tissue Samples

A total of 76 cases of HCCs tissues were obtained from patients collected by the Institute of General Surgical Research, Second Affiliated Hospital, Yangzhou University. In addition, 76 normal liver tissues were used as controls. Clinicopathological characteristics parameters are shown in Table 1. All of the HCC patients have not received previous treatments like local ablation, radiation therapy, chemoembolization, or chemotherapy.

### 2.2. Cell Culture

The human HCC-derived cell lines HepG2 were provided from the Medical Academy of Yangzhou University and cells were cultured in Dulbecco's modified essential medium (DMEM, Invitrogen Corp., USA). Both media were supplemented with 10% heat-inactivated fetal bovine serum (Zhejiang Tianhang Biotechnology Co., Ltd., China), penicillin, and streptomycin, at 37°C in a humidified incubator with 5% CO_2_. Cellular hypoxia environments were stimulated with 300 mM CoCl_2_ (Sigma-Aldrich Co. LLC., USA) for 24 h.

### 2.3. RT-qPCR

Total RNA from cell lines or tissue samples was extracted using a mirVana miRNA Isolation Kit (Ambion, Austin, TX, USA) following the manufacturer's instructions. The purity and concentration of RNA samples were assessed by standard spectrophotometric methods 2100 Bioanalyzer (Agilent Technologies, Santa Clara, CA). Briefly, 5 ng of RNA was added to RT reaction, and then the cDNA served as the template for amplification of PCR with sequence-specific primers (Sangon Biotech, Shanghai, China) using SYBR PrimeScript miRNA RT-PCR kit (Takara Biotechnology Co. Ltd., Dalian, China). All reactions were run in triplicate on the iCycler iQ Multicolor Real-Time PCR Detection System (BioRad, Hercules, CA, USA). Small nucleolar RNA GAPDH was used as an internal standard for normalization. Each sample was run in duplicate for analysis. The change for mRNA in HCC tissues relative to the matched normal liver tissues was calculated using the 2^−ΔΔCt^ method, where ΔΔCt = ΔCt HCC/normal liver tissues and ΔCt = Ct_miR-20b_ − Ct_GAPDH_.

For miR-20b, the primers were as follows: forward, 5′-TGTCAACGATACGCTACGA-3′ and reverse, 5′-GCTCATAGTGCAGGTAGA-3′; GAPDH forward, 5′-GTGGTCCAGGGTTTCTTACT-3′ and reverse, 5′-GTTGTCTCCTGCGACTTCA-3′; HIF-1*α* forward, 5′-AACGACAAGAAAAAGATAAGTTCT-3′ and reverse, 5′-GTTTGGTGTGGTTACATA-3′; VEGF forward, 5′-CAGGAACAAGGGCCTCTGTCT-3′ and reverse, 5′-TGTCCCTCTGACAATGTGCCATC-3′.

### 2.4. Transfection of miRNA

Transfection was performed using Lipofectamine 2000 (Invitrogen Life Technologies), in accordance with the manufacturer's instructions. For miRNA-20b functional analysis, the HepG2 cells were transfected with the scrambled miRNA as a negative control, miRNA-20b mimics, or miRNA-20b inhibitor (Ambion, Life Technologies, Grand Island, USA). For HIF-1*α* or VEGF functional analysis, the HepG2 cells were transfected with HIF-1*α* or VEGF-specific small interfering (si)RNA or pcDNA3.1-HIF-1*α* plasmid (Sangon Biotech, Shanghai, China). The transfection assay was performed as described in study [27].

### 2.5. Luciferase Reporter Gene Assay

The mRNA sequence targeted by the miRNA was predicted using TargetScan, miRanda, and NBmiRTar. The fragment was designated as HIF-1*α* 3′-UTR and inserted into pMIR-REPORTTM luciferase reporter vector (Sac I and Hind III restriction enzyme sites; Ambion, Life Technologies, Grand Island, USA). Another expressing vector was also constructed by the insertion of a mutated HIF-1*α* 3′-UTR using QuikChangeH Site-Directed Mutagenesis Kit (Stratagene, Santa Clara, CA). Then, the recombinant reporter vectors with normal and mutated HIF-1*α* 3′-UTR were cotransfected with miR-20b into HepG2 cells, respectively, using TransMessenger Transfection Reagent (Tiangen Biochemical Technology (Beijing) Co., Ltd., Beijing, China). The luciferase assay was performed according to the manufacturer's instructions.

### 2.6. Western Blot Analysis

Protein concentration was determined by BCA Protein Assay Kit (Santa Cruz, USA). About 30 *μ*g protein extracts were electrophoresed on 10% sodium dodecyl sulfate-polyacrylamide gel (SDS-PAGE), transferred onto PVDF membranes (BioRad Laboratories, Hercules, CA, USA), and incubated for 1 h in TBS containing 5% nonfat milk and 0.1% Tween-20 incubated overnight at 4°C with the following primary antibodies: anti-HIF-1*α* (1 : 1000, BD Transduction Laboratories, USA). After washing in TBS with 0.1% Tween-20, they were incubated for 1 h at room temperature with HRP-conjugated anti-rabbit antibody (1 : 1,000, Santa Cruz Biotechnology Inc., USA). Immunoreactivity was detected by enhanced chemiluminescence (ECL kit, Santa Cruz Biotechnology Inc., USA) and visualized by autoradiography. The level of *β*-actin (1 : 1,000, Santa Cruz Biotechnology Inc., USA) was used as a control of the amount of protein loaded into each lane and the optical density of each band was measured using ImageJ.

### 2.7. Viability Assay

For the cell viability assay, cells were seeded into a 96-well plate in quintuplicate; the cell growth was measured by CellTiter 96 AQueous One Solution Cell Proliferation Assay (MTS; Promega (Beijing) Biotechnology Co., Ltd., Beijing, China) after the indicated periods. Absorbance was measured at 540 nm using Versamax microplate reader (Molecular Devices, Sunnyvale, CA).

### 2.8. Annexin V-FITC/PI Determination of Apoptosis

Add precooled 70% ethanol to fixed cells. Take 1 mL of the cell suspension and add it to a centrifuge tube, centrifuge (4°C, 1000 r/min, 5 min). Use 1x binding buffer to adjust 1 × 10^6^/mL; take 100 *μ*L cell suspension to detect cell apoptosis, according to the Annexin V-FITC/PI kit (Nanjing Kaiji Biological Technology Development Co., Ltd., Nanjing, China) specification method steps.

### 2.9. Statistical Analysis

Data are presented as the mean ± standard deviation (SD). The significance of differences was evaluated by *t*-test. Differences with *P* values <0.05 were considered statistically significant. The postoperative survival rate was analyzed with the Kaplan-Meier method, and differences in survival rates were assessed with the log-rank test. All statistical analyses were performed using GraphPad Prism 6 software (GraphPad Software, San Diego, CA, USA).

## 3. Result

### 3.1. miR-20b Is Upregulated in HCC Tissues

**Figure 1:**
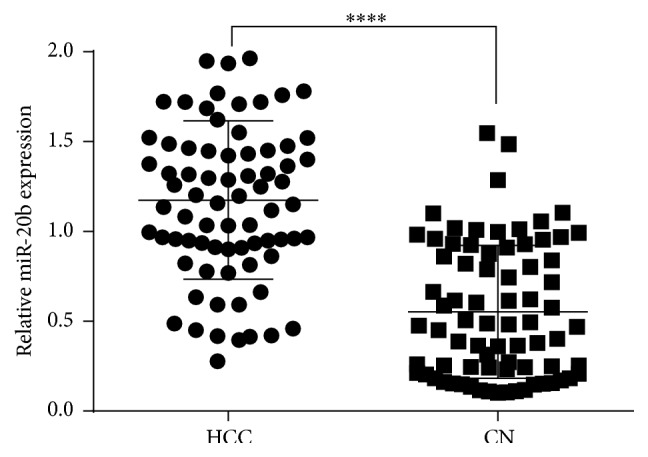
Comparison of miR-20b expression levels between HCC tissues and normal tissues (*P* = 0.000).

From the qRT-PCR result, we found that miR-20b expression levels were significantly upregulated in HCC cancer tissues compared to those in the normal control (*P* = 0.000, Figure 1).

Correlation between levels of miR-20b expression and clinicopathological characteristics of HCC patients was observed.

**Figure 2:**
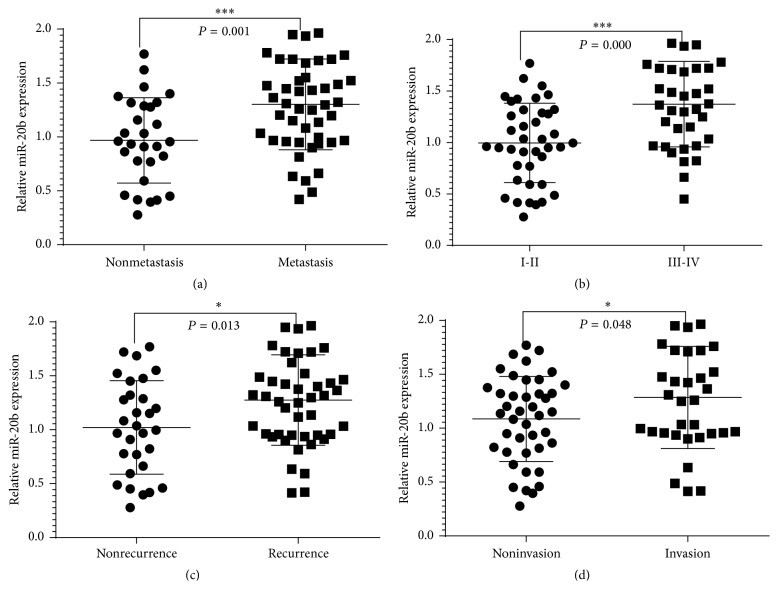
The correlation between levels of miR-20b and tumor metastasis, TNM stage, tumor recurrence, and microvascular invasion. (a) Tumor metastasis (*P* = 0.001), TNM stage (*P* = 0.000), tumor recurrence (*P* = 0.013), and microvascular invasion (*P* = 0.048).

The miR-20b expression levels were classified as high or low in relation to the median value. Meanwhile, we found the expression levels of miR-20b in HCC patients had no significant correlation with gender, age, tumor size, AFP (ng/mL), and tumor grade (*P* > 0.05; Table 1). But when comparing miR-20b expression in tumor metastasis, TNM stage, tumor recurrence, and microvascular invasion we found a significant difference (Figure 2).

### 3.2. miR-20b Expression and Postoperative Survival

**Figure 3:**
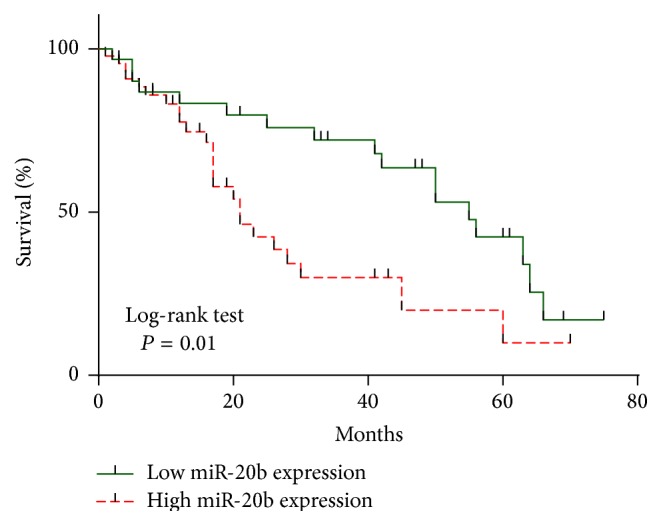
Kaplan-Meier survival curve of HCC patients. Patients in the high expression group had significantly poorer prognosis than those in low expression group, analyzed by log-rank tests which are indicated (*P* = 0.01).

Kaplan-Meier survival curve and log-rank test for 76 patients with HCC with high expression or low expression of miRNA-20b in tumor tissue were analyzed. The overall survival (OS) rate of HCC patients was significantly lower with high miR-20b mRNA expression than that in those with low expression; it means that high miR-20b expression might be correlated with poor prognosis of HCC patients (*P* = 0.01; Figure 3). We also use univariate and multivariate Cox model analyses which were performed to determine the correlation of miR-20b expression with overall survival of HCC patients. In univariate analysis the found metastasis, TNM stage, tumor recurrence, microvascular invasion, and miR-20b were statistically significant prognosis factors (Table 2). A multivariate analysis confirmed that metastasis, TNM stage, microvascular invasion, and miR-20b expression were significant independent predictors of poor survival of HCC (Table 3).

### 3.3. Inverse Level of miR-20b and HIF-1*α*, VEGF in HepG2 Cells under Normal or Hypoxia-Mimetic Conditions

**Figure 4:**
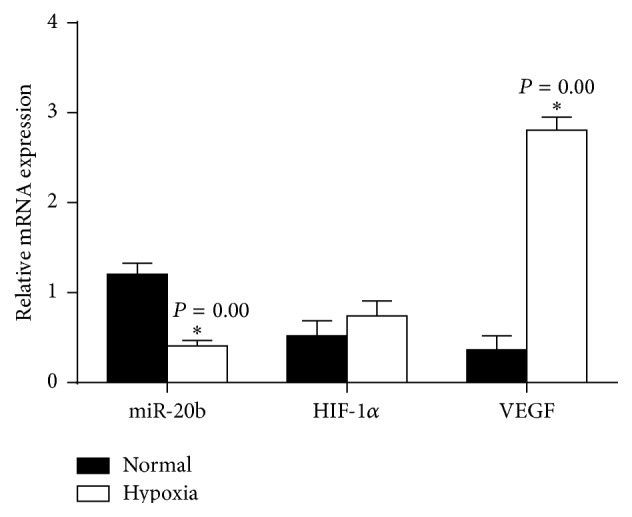
The levels of miR-20b and HIF-1*α*, VEGF expression after treatment of CoCl_2_ for 24 h. ^*∗∗*^ *P* < 0.01 compared with normal group.

**Figure 5:**
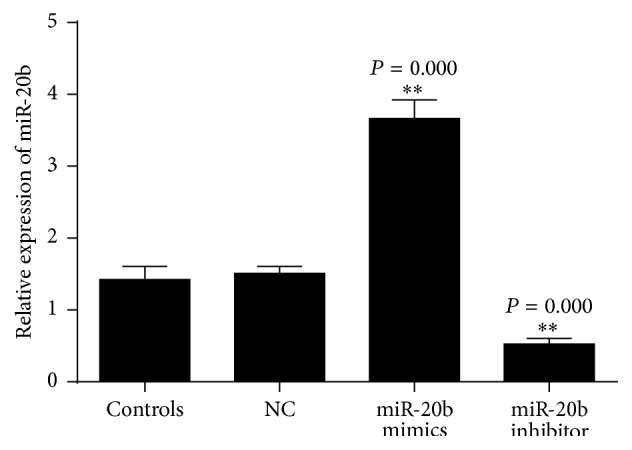
The transcription efficiency was performed to determine the levels of miR-20b transfected with scrambled miRNA (NC), miR-20b mimics, and miR-20b inhibitor. ^*∗∗*^ *P* < 0.05 compared with control.

To further investigate the role of miR-20b in the regulation of HIF-1*α*, VEGF expression in normal and hypoxia-mimetic conditions was analyzed. We used CoCl_2_ to mimic hypoxia conditions, the cells treatment of CoCl_2_ for 24 h. The levels of miR-20b and HIF-1*α*, VEGF expression in normal and hypoxic conditions were analyzed (Figure 4). Then the HepG2 cells were transfected with scrambled miRNA, miR-20b mimics, and miR-20b inhibitor, respectively. The transfection efficiency is as shown in Figure 5.

### 3.4. HIF-1*α*, VEGF Are Direct Targets of miR-20b

**Figure 6:**
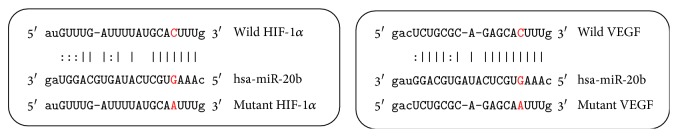
miR-20b targeted 3′-UTR of HIF-1*α*, VEGF mRNA.

**Figure 7:**
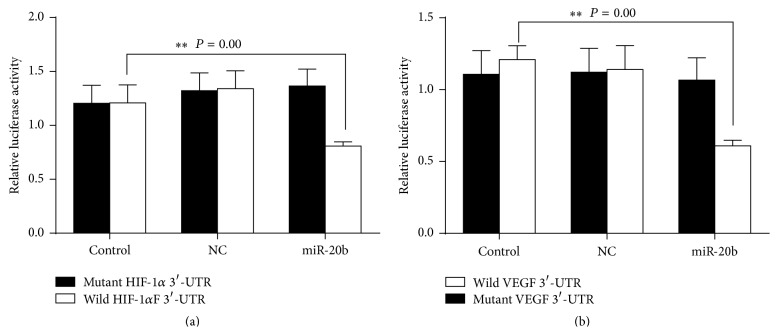
Luciferase reporter assay in HepG2 cells. Cells were cotransfection of HepG2 cells with miR-20b mimics and wild-type HIF-1*α*, VEGF 3′-UTR led to a marked decrease in luciferase activity and cotransfection with miR-20b and mutant HIF-1*α*, VEGF 3′-UTR had no effect on luciferase activity, and cotransfection with NC miRNA and wild-type HIF-1*α*, VEGF 3′-UTR or mutant HIF-1*α*, VEGF 3′-UTR also showed no difference, ^*∗∗*^ *P* < 0.05 compared with negative control.

**Figure 8:**
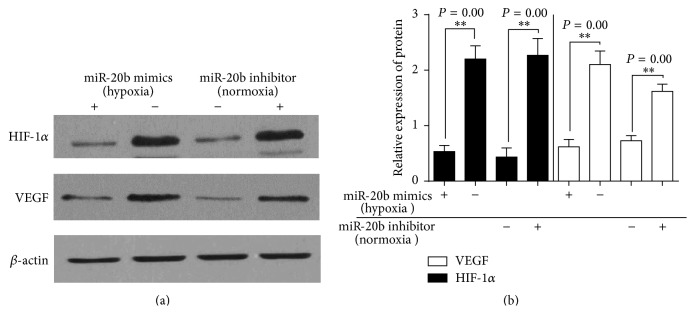
In hypoxia group showing HIF-1*α*, VEGF decreased after miR-20b treated transfected. In normal group miR-20b inhibitor increased HIF-1*α*, VEGF protein by Western blot (a). Use ImageJ to analyze relative expression of proteins (b). ^*∗∗*^ *P* < 0.05 compared with each control group.

Bioinformatical predication was performed using TargetScan, miRanda, and NBmiRTar to predict targets of miR-20b. We found miR-20b at the 3′-UTR of HIF-1*α*, VEGF which were highly conserved (Figure 6). To verify whether HIF-1*α*, VEGF were direct targets of miR-20b, the wild and mutant types of HIF-1*α*, VEGF 3′-UTR were generated. The dual-luciferase reporter assay was subsequently performed in hepatocellular carcinoma HepG2 cells. As shown in Figures 7(a) and 7(b), the luciferase activity was significantly reduced in HepG2 cells cotransfected with the wild-type 3′-UTR of HIF-1*α*, VEGF and miR-20b mimics but unchanged in HepG2 cells cotransfected with the mutant HIF-1*α*, VEGF 3′-UTR and miR-20b mimics, indicating that miR-20b directly binds to the 3′-UTR of HIF-1*α*, VEGF in HepG2 cells. We transfected miR-20b mimics and miR-20b inhibitor to HepG2 cells. The miR-20b inhibitor was transfected into normoxic cells; miR-20b mimics were transfected into hypoxic cells and the HIF-1*α*, VEGF expression detected by Western blot (Figure 8).

### 3.5. miR-20b Enhances HepG2 Cell Proliferation

**Figure 9:**
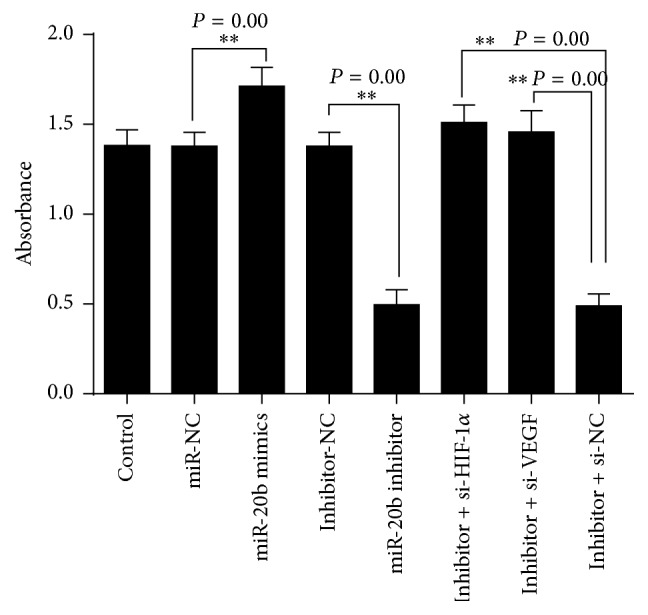
The cells viability detected by MTS assays. HepG2 cells were transfected with miR-20b mimics, miR-20b inhibitor, inhibitor + si-HIF-1*α*, and inhibitor + si-VEGF. Inhibitor + si-HIF-1*α*: miR-20b inhibitor + HIF-1*α*-siRNA; inhibitor + si-VEGF: miR-20b inhibitor + VEGF-siRNA. ^*∗∗*^ *P* < 0.01 compared with control.

The MTS assay was used to investigate the miR-20b effects cell viability. We transfected miR-20b mimics into cells; the cell viabilities significantly increased after transfected miR-20b mimics for 24 h in normoxia comparison with that of negative control (miR-NC) (Figure 9; ^*∗∗*^ *P* < 0.01). We also transfected miR-20b inhibitor into cells in normoxia; the cell viabilities were dramatically restrained compared with that of negative control (inhibitor-NC) transfected cells (Figure 9; ^*∗∗*^ *P* < 0.01), to verify the ways miR-20b affects tumor cells growth. Furthermore we also transfected inhibitor + si-HIF-1*α* or inhibitor + si-VEGF into cells. When the cells were cotransfected with miR-20b inhibitor and HIF-1*α*-siRNA or VEGF-siRNA the cell viabilities significantly increased compared with only transfected miR-20b inhibitor (Figure 9; ^*∗∗*^ *P* < 0.01).

### 3.6. Downregulation of miR-20b Enhanced the Resistance to Apoptosis

**Figure 10:**
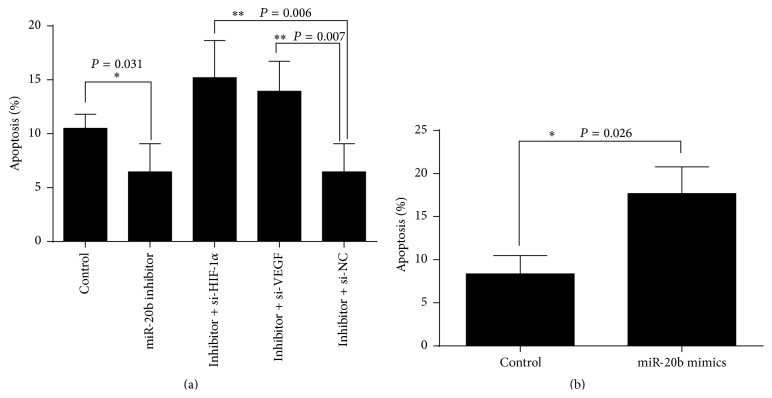
The cells apoptosis detected by Annexin V-FITC/PI assay. HepG2 cells were transfected with miR-20b inhibitor, inhibitor + si-HIF-1*α*, and inhibitor + si-VEGF. Inhibitor + si-HIF-1*α*: miR-20b inhibitor + HIF-1*α*-siRNA; inhibitor + si-VEGF: miR-20b inhibitor + VEGF-siRNA. ^*∗∗*^ *P* < 0.01 compared with control in normoxia (a). Transfected miR-20b mimics for 24 h in mimetic hypoxia conditions; apoptosis significantly increased in hypoxia conditions (b).

The Annexin V-FITC/PI assay was used to investigate the cell apoptosis effects of miR-20b. We transfected miR-20b inhibitor into cells; the cell apoptosis significantly decreased in comparison with that of control in normal environment (Figure 10(a); ^*∗*^ *P* < 0.05). Furthermore we also transfected inhibitor + si-HIF-1*α* or inhibitor + si-VEGF into cells for 24 h in normoxia. When the cells were cotransfected with miR-20b inhibitor and HIF-1*α*-siRNA or VEGF-siRNA the cell apoptosis significantly increased compared with only transfected control group (Figure 10(a); ^*∗∗*^ *P* < 0.01). By the way, we transfected miR-20b mimics for 24 h and used CoCl_2_ to mimetic hypoxia conditions; the cells treatment of CoCl_2_ for 24 h was then detected by flow cytometry. Comparison with control group transfected miR-20b mimics the cells apoptosis significantly increased (Figure 10(b); ^*∗*^ *P* < 0.05).

## 4. Discussion

In the present study, many miRNAs have confirmed contribution to the initiation and progression of HCC. With further in-depth studies more and more miRNAs have shown that take part in the regulation of hepatocellular carcinoma development. Furthermore, it has been shown that miRNAs can function as tumor suppressors or oncogenes and repress the expression of important cancer-related genes and might prove useful biomarkers in the diagnosis and treatment of cancers [35]. The important role in hepatocellular carcinoma tells us that understanding of miRNA function will provide us with broad prospects to understand and overcome tumor in the future.

miR-20b belongs to the miR-106a-363 cluster, which together with miR-17-92 and miR-106b-25 clusters forms a large family of highly similar miRNAs called the miR-17 family [28]. In the present study, the high expression levels of miR-20b often promote tumor development so the miR-20b suggested can serve as a potential oncogene. In our study we found that miR-20b expression levels were significantly upregulated in HCC cancer tissues compared to those in the normal control (*P* = 0.000, Figure 1). Furthermore, miR-20b expression showed significant association with tumor metastasis, TNM stage, tumor recurrence, and microvascular invasion comparison of clinicopathological factors which is an important clinical determinant for the prognosis of HCC patients. In Kaplan-Meier survival curve analysis, OS rates of HCC patients rates indicated that with high miR-20b expression there was significantly poorer survival in comparison with low miR-20b expression. In a multivariate Cox model, we found that high miR-20b expression was an independent factor for predicting the 5-year OS of HCC patients. From clinicopathological characteristics study we found that the expression of miR-20b was upregulated in HCC. High expression of miR-20b was significantly associated with tumor progression and decreased OS in patients of HCC indicating that it might play critical roles in HCC progression and development.

Based on our clinicopathological characteristics study, we further investigated the function and possible mechanisms of miR-20b in regulating some biological properties of HepG2 cells. We confirmed that miR-20b binds with the HIF-1*α* and VEGF 3′-UTR by luciferase reporter assay. Under normoxic conditions, posttranslational HIF-1*α* is rapidly degraded by the proteasome and usually not detectable and HIF-1*α* is a mediator of cell response to hypoxia in mammalian cells cultured under reduced oxygen tension [24]. Among the target genes of HIF-1*α*, vascular endothelial growth factor (VEGF) is one of the major target genes for HIF-1*α* that directly participates in angiogenesis [36]. We used CoCl_2_ in mimetic hypoxia conditions and found inverse level of miR-20b and HIF-1*α*, VEGF in HepG2 cells under normal or hypoxia-mimetic conditions. From the result we found that in hypoxia group miR-20b expression significantly decreased and VEGF mRNA significantly increased; the hypoxia conditions are not to change HIF-1*α* mRNA expression but significantly increase HIF-1*α* protein. To verify relationship between miR-20b and HIF-1*α*, VEGF expression, we also transfected miR-20b mimics and miR-20b inhibitor to HepG2 cells. The miR-20b inhibitor was transfected into normoxic cells; miR-20b mimics were transfected into hypoxic cells and the HIF-1*α*, VEGF expression detected by Western blot. As a result we found HIF-1*α* and VEGF are targets by miR-20b. In normal condition HepG2 highly expresses miR-20b but reduces HIF-1*α*, VEGF. Interestingly, once we put the cells in hypoxia-mimetic conditions the cell response to hypoxia would highly express HIF-1*α*, VEGF and then reduced levels of miR-20b.

In present studies reported, miR-20b expression could affect cell biological behaviour [28, 31, 32, 34, 37]. We used the loss-of-function and gain-of-function approaches which showed that miR-20b could impact the cell viability and apoptosis. The miR-20b mimics and miR-20b inhibitor were transfected into HepG2 cells in normoxic condition. In miR-20b mimics group, the cells viability significant increased compared with control group. While in miR-20b inhibitor group the cells viability decreased significantly, at some time we cotransfected miR-20b inhibitor and HIF-1*α*-siRNA or VEGF-siRNA; the cells proliferative capacity has been enhanced. The result tells us that miR-20b maintains tumor cell growth through its regulation of HIF-1*α* and VEGF in normal condition. The cells apoptosis result also suggests that high expression of HIF-1*α* and VEGF in normal condition would help cells to resist apoptosis. Considering the regulation of apoptosis genes by HIF-1*α* and VEGF [38, 39], when in hypoxia-mimetic conditions we transfected miR-20b mimics into cells; it is means that we also decreased levels of HIF-1*α* and VEGF which lead to cells apoptosis raised.

In conclusion, in the present study we report that miR-20b is associated with poor overall survival of HCC patients, suggesting its potential prognostic values in this disease type, and identified HIF-1*α* and VEGF as direct targets of miR20b in hepatocellular carcinoma cells. From our cells studies we found not only miR-20b regulation of HIF-1*α* and VEGF in normal but also HIF-1*α* and VEGF regulation of miR-20b in hypoxia. This mechanism would help the tumor cells adapt to the different environments thus promoting the tumor invasion and development.

## Figures and Tables

**Table 1:** Association between miR-20b expression and clinicopathological features of HCC.

Variables	miRNA-20b expression high (*n* = 45)	miRNA-20b expression low (*n* = 31)	*P*
Age (years)			
≤57	20	18	0.243
>57	25	13	
Gender			
Male	32	25	0.356
Female	13	6	
Tumor size (cm)			
≥5	27	15	0.317
<5	18	16	
AFP (ng/mL)			
≥400	19	17	0.279
<400	26	14	
Metastasis			
Yes	37	10	0.000^*∗*^
No	8	21	
Tumor grade			
G1	12	6	0.147
G2	17	7	
G3	16	18	
TNM stage			
I-II	14	26	0.000^*∗*^
III-IV	31	5	
Tumor recurrence			
Yes	32	14	0.023^*∗*^
No	13	17	
Microvascular invasion			
Yes	29	5	0.016^*∗*^
No	16	26	

^*∗*^ *P* < 0.05.

**Table 2:** Univariate analysis of clinicopathological factors for overall survival.

Variable	*n*	Hazard ratio	95% CI	*P* value
Age (years)				
≤57	38	1	0.866–1.417	0.350
>57	28	0.728		
Gender				
Male	57	1	0.912–3.749	0.088
Female	19	1.849		
Tumor size (cm)				
≥5	42	1	0.264–1.116	0.097
<5	34	0.543		
AFP (ng/mL)				
≥400	36	1	0.387–1.506	0.436
<400	40	0.434		
Metastasis				
Yes	47	1	1.396–5.647	*0.004* ^*∗*^
No	29	2.808		
Tumor grade				
G1	18	1	0.710–2.751	0.333
G2	24	1.398		
G3	34			
TNM stage				
I-II	40	1	1.626–6.946	*0.001* ^*∗*^
III-IV	36	3.360		
Tumor recurrence				
Yes	46	1	0.171–0.657	*0.007* ^*∗*^
No	30	0.360		
Microvascular invasion				
Yes	34	1	1.187–5.931	*0.017* ^*∗*^
No	42	2.654		
miR-20b				
High	45	1	0.129–0.528	*0.000* ^*∗*^
Low	31	0.261		

^*∗*^ *P* < 0.05.

**Table 3:** Multivariate analysis of clinicopathological factors for overall survival.

Variable	*n*	Hazard ratio	95% CI	*P* value
Metastasis				
Yes	47	0.295	0.124–0.696	*0.005* ^*∗*^
No	29			
TNM stage				
I-II	40	5.031	1.627–15.558	*0.005* ^*∗*^
III-IV	36			
Tumor recurrence				
Yes	46	1.418	0.522–3.854	0.493
No	30			
Microvascular invasion				
Yes	34	0.215	0.088–0.527	*0.001* ^*∗*^
No	42			
miR-20b				
High	45	5.018	2.325–11.223	*0.000* ^*∗*^
Low	31			

^*∗*^ *P* < 0.05.
